# Recurrent Serous Borderline Tumor After Fertility-Sparing Surgery Following Twin Pregnancy and Resumption of Menstruation

**DOI:** 10.7759/cureus.52296

**Published:** 2024-01-15

**Authors:** Noriko Morita, Hiroshi Matsushita, Hiromitsu Yabushita, Akihiko Wakatsuki

**Affiliations:** 1 Department of Obstetrics and Gynecology, Aichi Medical University, Nagakute, JPN

**Keywords:** surgical menopause, borderline ovarian tumor, recurrence, hormone replacement therapy, fertility-sparing surgery, estrogen

## Abstract

Gynecologic malignancies sometimes affect women before menopause. Aggressive treatments, such as surgery, chemotherapy, and/or radiotherapy, often lead to premature menopause. Hormone replacement therapy (HRT), typically used for managing menopause-associated health issues, may be limited by tumor sensitivity to estrogen. Here, we present a case of a 37-year-old woman seeking fertility, who was diagnosed with a serous borderline ovarian tumor (BOT). Fertility-preserving surgery and in-vitro fertilization resulted in a twin pregnancy. During a postpartum amenorrheic period, there was no recurrence. However, she experienced a rapid recurrence of the disease following the resumption of menstruation and underwent radical surgery. This rapid recurrence after menstruation resumed suggests potential estrogen sensitivity. Close postoperative monitoring has been ongoing without HRT.

## Introduction

Approximately 30-40% of women diagnosed with gynecologic cancer are in the pre- or perimenopausal stage [1]. Given that the standard treatment for gynecologic cancers typically involves surgery, chemotherapy, and/or radiotherapy, these therapies often result in the loss of ovarian function and induced menopause. Nevertheless, fertility-sparing surgery (FSS) may be an option for women aiming to preserve fertility [2], especially if their disease is in the early stage, low-grade or chemo-sensitive. In cases where radical therapies including bilateral oophorectomy, hormone replacement therapy (HRT) is recommended to alleviate symptoms and sequelae associated with menopause [3].

Borderline ovarian tumors (BOTs) are characterized by epithelial proliferation and atypia without stromal invasion, constituting 10-20% of all epithelial ovarian tumors [2]. BOTs tend to be diagnosed at significantly earlier stages and younger ages compared to invasive ovarian cancer, making fertility preservation a primary concern for affected women who have not completed childbearing [2]. A systematic review encompassing 2,479 BOT patients demonstrated that 923 (37%) underwent FSS, with 103 (48%) of 213 attempting to conceive becoming pregnant [4]. While the recurrence rates post FSS (23.8-66.7%) are higher than those post radical surgery (5.4-30.8%) in advanced-stage BOT patients [5], FSS remains a viable option as subsequent surgeries for recurrent tumors enhance the overall prognosis [6].

In this report, we present a case of a patient diagnosed with stage IIIA serous BOT who achieved conception through in-vitro fertilization and embryo transfer (IVF-ET) following FSS. Unfortunately, the patient underwent radical surgery due to a recurrence soon after the resumption of menstruation. This case highlights that an elevation in endogenous estrogen level may be a risk of serous BOT recurrence after FSS.

The abstract of this article was previously presented as a poster at the 74th Annual Congress of Japan Society of Obstetrics and Gynecology in Fukuoka, Japan, on August 5-7, 2022.

## Case presentation

**Figure 1:**
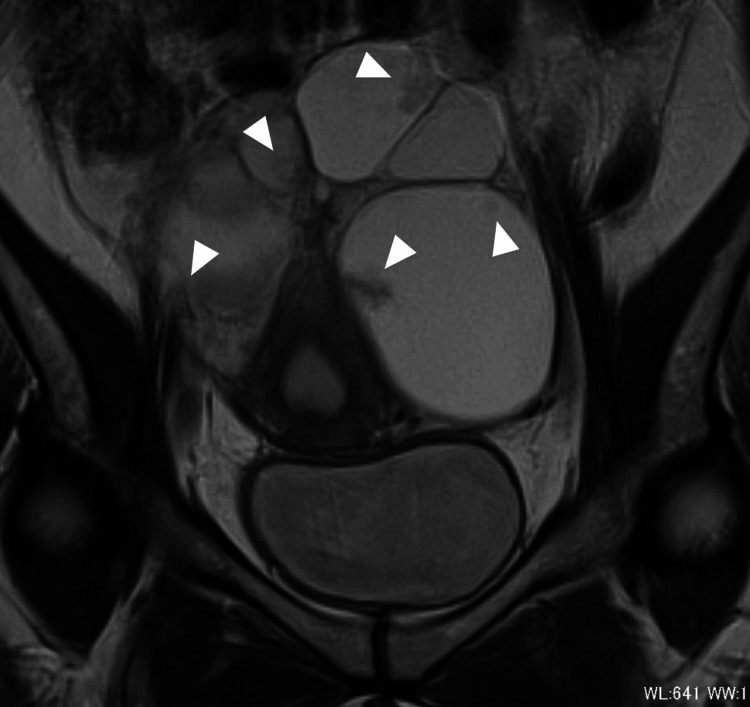
Preoperative T2-weighted magnetic resonance imaging scan (coronal section) showing bilateral multiloculated ovarian tumors with intracystic papillary projection (arrowheads).

A 37-year-old nulligravid Japanese women consulted a gynecologist due to infertility issues persisting over five years. During the infertility workup, a pelvic mass was detected. Magnetic resonance imaging revealed bilateral multiloculated ovarian tumors (left: 125 × 88 mm; right: 50 × 35 mm) with intracystic papillary projections (Figure 1). A pelvic CT scan indicated scattered tiny nodules, suggesting peritoneal dissemination. These findings led to a suspicion of ovarian cancer, promoting referral to explore fertility-sparing surgical options.

**Figure 2:**
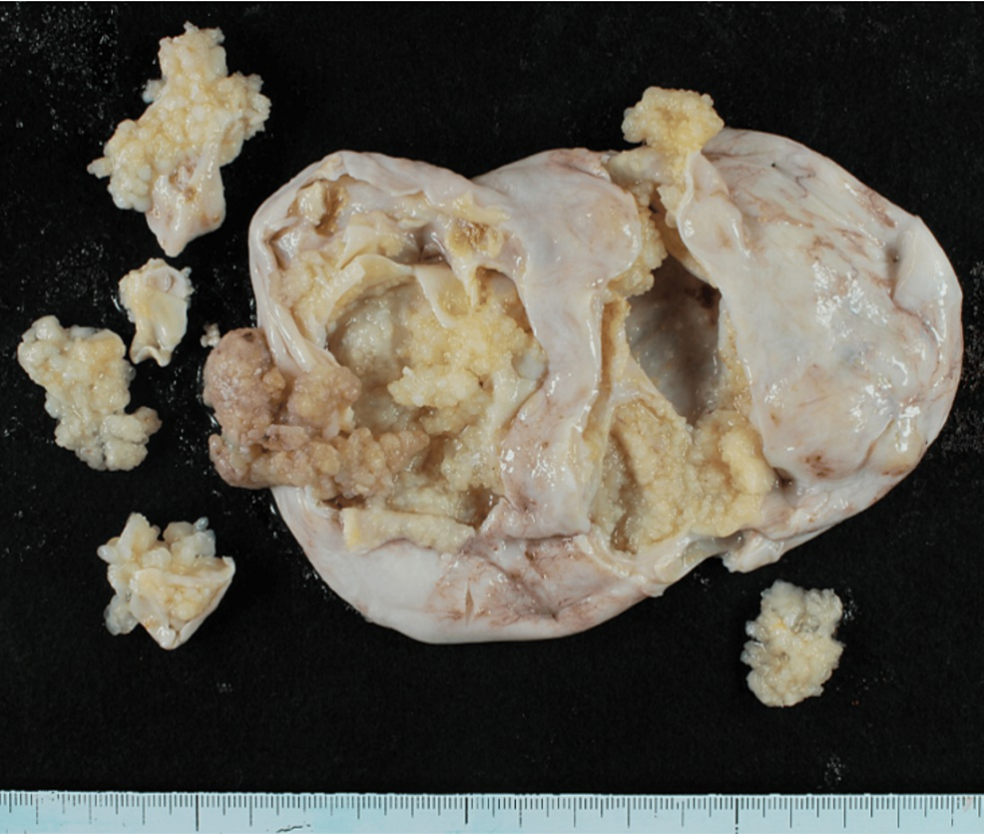
Photographs of the left ovary showing a multinodular mass with multiple intracystic nodules.

**Figure 3:**
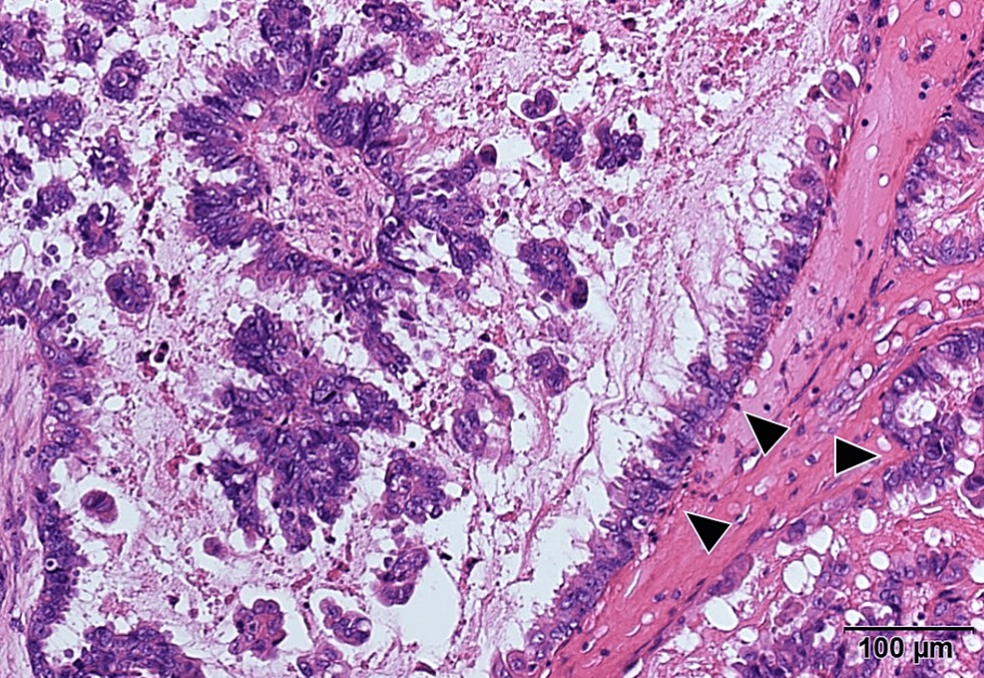
Histological analysis of the left ovarian mass showed epithelial proliferation and atypia without stromal invasion (arrowheads) (H&E staining).

Her serum CA125 level was elevated at 309 U/mL, and CA19-9 remained within normal limits. Her past medical history was unremarkable. Subsequently, she underwent an exploratory laparotomy, which revealed bilateral ovarian tumors. Frozen section analyses of these masses strongly indicated serous BOTs. Additionally, fine nodules indicative of dissemination were observed on the surface of the right ovary, uterus, and peritoneum, which were compatible with the dissemination of the serous BOTs. Consequently, she underwent FSS, which included a left salpingo-oophorectomy, resection of the right ovarian tumor, and samplings of the peritoneum and omentum (Figure 2). Examination of permanent histopathological specimens confirmed the bilateral ovarian tumors as serous BOTs (Figure 3). Histopathologically, the dissemination observed on the omentum, right ovary, and peritoneum lacked desmoplastic changes and were interpreted as non-invasive implants. As per the FIGO Ovarian Cancer Staging Guidelines (2014), she was diagnosed with stage IIIA2 serous BOT.

After her surgery, she underwent intracytoplasmic sperm injection and frozen embryo transfer at an infertility clinic, leading to a dizygotic twin pregnancy. Two years post-surgery, female infants weighing 2,166 and 1,926 g were delivered via cesarean section at 34 weeks’ gestation. During the cesarean section, the right ovary appeared normal in size, and there were no signs indicating recurrence or implants. For over a year post-surgery, the patient experienced amenorrhea without any disease recurrence.

**Figure 4:**
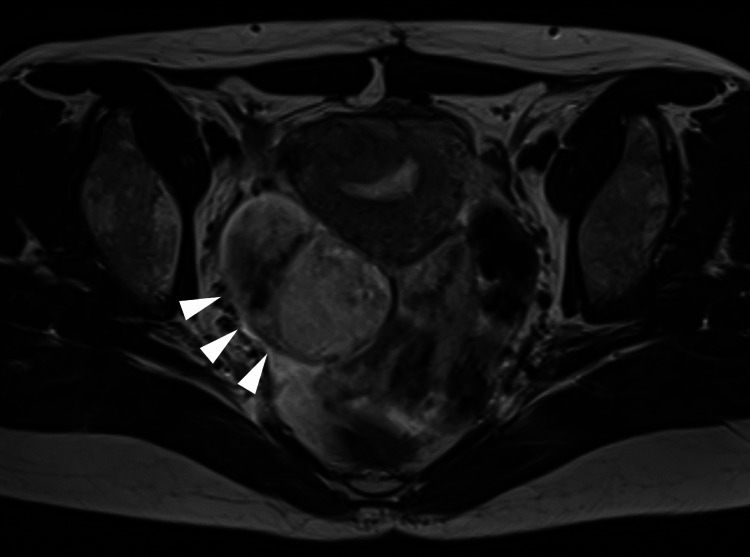
T2-weighted magnetic resonance imaging scan (transverse section) showing right ovarian tumor, suggesting the recurrence of serous borderline tumor (arrowheads).

However, a month after the resumption of menstruation, ultrasonography during her first follow-up visit revealed a 4.1 × 3.9 cm right ovarian cyst with intracystic papillary projections. Subsequent laparotomy confirmed the presence of serous BOT, interpreted as a recurrence (Figure 4). Consequently, she underwent a right salpingo-oophorectomy along with a total hysterectomy and omentectomy, resulting in surgical menopause. Immunohistochemically, the tumor cells were positive for estrogen and progesterone receptors. As of 1.5 years post the second surgery, the patient’s postoperative course has shown no evidence of disease recurrence.

## Discussion

In this report, we present a case of serous BOT treated with FSS. Despite successfully giving birth to twins through IVF-ET, the patient faced recurrence and subsequent surgical menopause following radical surgery. Compared to natural menopause, surgical menopause causes a rapid decline in systemic estrogen levels, leading to short-term consequences, such as heightened vasomotor symptoms, increased rates of mood disorders, sleep disturbances, sexual dysfunction, joint symptoms, and reduced quality of life [3]. Studies such as the Nurses’ Health Study indicated that women undergoing bilateral oophorectomy before age 50 have an elevated risk of all-cause mortality, coronary heart disease (CHD), and stroke [7]. However, recent research has shown that women who experience surgical menopause before 50, and using HRT, exhibit a lower risk of incident CHD compared to non-users of HRT [8].

To mitigate sequelae following surgical menopause, immediate introduction of HRT is recommended post-surgery until the average age of natural menopause (around 51 years) unless contraindications to HRT are present. Contraindications may include vaginal bleeding, liver disease, prior history of CHD, stroke, myocardial infarction, venous thromboembolism, or a personal or inherited high risk of thromboembolic disease [9]. While a historyof gynecological cancer is not an absolute contraindication for HRT, its use in women with such a history of gynecological cancer should be determined individually, considering the estrogen sensitivity of the tumor. However, the Society of Gynecologic Oncology clinical practice statement reported that HRT is not recommended in women with a history of BOT due to data paucity [10].

While the precise impact of HRT on the pathogenesis of BOTs remains unclear, a limited number of studies suggest a possible association between past or present HRT usage and a higher likelihood of developing a serous BOT. Mills et al. [11] conducted a study involving 74 cases of BOTs (55 serous and 19 mucinous) and found that women using HRT for a short duration (two to three years) faced an elevated risk of developing a BOT. Similarly, Rasmussen et al. [12] reported a more than 30% increased risk of developing a serous BOT.

Moreover, Riman et al. [13] noted an augmented risk of serous BOT following unopposed estrogen therapy, although no such risk was observed with estrogen supplemented by either cyclic or continuous progestin. However, regarding the potential impact of HRT on BOT recurrence, a retrospective descriptive study by Mascarenhas et al. reported no recurrent cases among 72 women who used HRT after a BOT diagnosis [14]. Considering these findings, the guideline from the French national network cautiously recommends the prescription of HRT after serous BOT diagnosis, particularly in cases without high-risk criteria, such as micropapillary pattern, stromal invasion, and peritoneal implants [15].

Uehara et al. [16] recently documented a case of pelvic seromucinous borderline tumor recurring 26 years after the resection of seromucinous BOT, followed by 10 years of HRT. This case emphasizes the need for physicians to remain vigilant regarding disease recurrence post-surgery, particularly in cases where HRT was administered for endometriosis-related ovarian neoplasms (ERONs). Although serous BOT is not typically classified as an ERON, unlike most BOT histotypes (endometrioid, clear cell, and seromucinous), there are indications that estrogen might contribute to serous BOT recurrence. Firstly, FSS has been identified as a significant risk factor for serous BOT recurrence, occurring in 25.0-56.4% of patients who undergo FSS [17]. Additionally, instances of serous BOT associated with pregnancy have been reported in the literature [18,19]. For instance, Matsumoto et al. [19] detailed a case of serous BOT diagnosed at seven weeks of gestation. Although the patient remained recurrence-free for four years, the recurrent disease was diagnosed at 13 weeks of her subsequent pregnancy. Similarly, Cosentino et al. [20] reported a case of recurrent serous BOT during pregnancy, occurring a year after tumor resection during a cesarean section.

Although the precise mechanisms underlying the involvement of estrogen in the recurrence of serous BOT remain unclear, the unusual occurrence of recurrent serous BOT emerging shortly after the postpartum amenorrheic period hints at a potential association between estrogen and tumor recurrence. Consequently, the patient has been meticulously monitored postoperatively without HRT.

## Conclusions

Physicians should remain vigilant regarding the possibility of serous BOT recurrence in scenarios where endogenous estrogen levels are elevated. This case prompts the need for further studies to delve into the influence of estrogen on the recurrence of serous BOT, aiming to enhance the quality of life for women following surgical menopause. Understanding this relationship could significantly contribute to better patient care and management.
